# PD-1 axis expression in musculoskeletal tumors and antitumor effect of nivolumab in osteosarcoma model of humanized mouse

**DOI:** 10.1186/s13045-018-0560-1

**Published:** 2018-02-06

**Authors:** Bingxin Zheng, Tingting Ren, Yi Huang, Kunkun Sun, Shidong Wang, Xing Bao, Kuisheng Liu, Wei Guo

**Affiliations:** 1 Musculoskeletal Tumor Center, Peking University People’s Hospital, No. 11 Xizhimen South Street, Beijing, 100044 People’s Republic of China; 2 Beijing Key Laboratory of Musculoskeletal Tumor, Beijing, People’s Republic of China; 3 Department of Pathology, Peking University People’s Hospital, Beijing, People’s Republic of China

**Keywords:** Osteosarcoma, PD-1/PD-L1/PD-L2, Prognosis, Humanized mouse, Nivolumab

## Abstract

**Background:**

Immune checkpoint inhibitors have led to a breakthrough in solid tumor immunotherapy, but related studies on musculoskeletal tumors are few, especially for PD-L2.

**Methods:**

We examined expression of three molecular effectors of the PD-1 axis in 234 patients with musculoskeletal tumors, including osteosarcoma, chondrosarcoma, synovial sarcoma, and giant cell tumor. Survival analyses and potential mechanisms were investigated in osteosarcoma per the Gene Expression Omnibus (GEO) and immunohistochemistry analyses. In vivo, humanized mice were used to evaluate the effect of nivolumab on osteosarcoma.

**Results:**

PD-L1, PD-L2, and PD-1 expression levels were significantly different between the histologic types of the musculoskeletal tumors. For osteosarcoma, PD-L1 was negatively correlated with prognosis, while PD-1 had a negative correlation tendency with overall survival (OS). Meanwhile, PD-L2 had a positive correlation trend with OS. Nivolumab inhibited osteosarcoma metastasis in humanized mice by increasing CD4+ and CD8+ lymphocytes and the cytolytic activity of CD8 lymphocytes in the lung but did not affect primary osteosarcoma growth.

**Conclusion:**

We systematically detected the expression patterns of PD-L1, PD-L2, and PD-1 in musculoskeletal tumors for the first time and demonstrated the prognostic roles and underlying mechanisms of PD-1 axis in osteosarcoma. Furthermore, PD-1 blockade could effectively control osteosarcoma pulmonary metastasis in vivo. Therefore, the PD-1 axis may be a potential immunotherapeutic target for metastatic osteosarcoma.

**Electronic supplementary material:**

The online version of this article (10.1186/s13045-018-0560-1) contains supplementary material, which is available to authorized users.

## Background

Sarcomas, characterized by high heterogeneity, are the main types of malignant bone and soft-tissue tumors [1], and neoadjuvant chemotherapy combined with surgery is the mainstream treatment strategy for most sarcomas. However, chemotherapy for sarcomas has entered the platform stage, and more than 40% of sarcoma patients ultimately experience tumor metastasis or recurrence with a poor prognosis [2]. Moreover, when traditional treatment fails for aggressive disease, few treatment choices are left. However, some sarcomas, such as chondrosarcoma, are not sensitive to chemotherapy or radiotherapy, and surgery is the only effective treatment. Therefore, when the tumor metastasizes or cannot be resected, both the patients and doctors are faced with a devastating dilemma. Therefore, novel and effective therapies for sarcomas are urgently needed to mitigate these desperate situations.

PD-L1 and PD-L2 are both ligands of PD-1, and these interactions transduce co-inhibitory signals for T cell activation, suppress T cell function, which is called T cell exhaustion, and ultimately promote tumor evasion of the immune system [3, 4]. For the past few years, immune checkpoint inhibitors (ICIs) have led to a breakthrough in solid tumor immunotherapy by relieving the immunosuppressive state of the tumor microenvironment and restoring the immune function of T cells to kill tumor cells [5–7]. Previous studies have shown that PD-1, PD-L1, and PD-L2 have different prognostic roles in various tumors [8–19]. The combination between PD-L2 and PD-1 also has a negative effect on T cell activation, which may be an important reason why some tumors express little to no PD-L1 yet still respond to PD-1 immunotherapy. This observation has aroused the interest of numerous scholars. However, current studies on sarcoma immunotherapy are limited, and the existing studies mainly focus on PD-L1 or PD-1 expression and their clinical implications in sarcomas [20–27]. To our knowledge, there have been no studies of PD-L2 expression in sarcomas.

In our study, we systematically investigated the expression patterns of PD-L1, PD-L2, and PD-1 in sarcomas including osteosarcoma, chondrosarcoma, synovial sarcoma, and giant cell tumors (GCTs) and further evaluated the association between PD-L1, PD-L2, and PD-1 expression and clinical prognosis of osteosarcoma to provide a therapeutic strategy guide. Then, we investigated the therapeutic effect of nivolumab on osteosarcoma and its underlying mechanism.

## Methods

### Tissue microarray construction

Three tissue microarray (TMA) slides were used to evaluate the expression patterns of the PD-1 axis. One was constructed using samples acquired from the Musculoskeletal Tumor Center, Peking University People’s Hospital (Beijing, China), and the relevant tumor tissues, including osteosarcoma (62 cases) and dedifferentiated chondrosarcoma (4 cases), were acquired at the time of definitive surgery and disease recurrence (either local or metastatic) with several paired samples included on the array. Core tissue (3 mm in diameter) was obtained from each donor block and placed in the recipient tissue array block. TMA sections (5-μm thickness) were sliced and preserved properly at room temperature for subsequent experiments. Informed consent was obtained from each patient, and the study was approved by the ethics committee of Peking University People’s Hospital. Clinical and histopathologic data were collated through a retrospective review of patient records. The other two TMAs (OS803 and SS1501) were purchased from US Biomax, Inc. Among them, the SS1501 TMA included chronic synovitis (9 cases), giant cell tumor (14 cases), and synovial sarcoma (127 cases); the OS803 TMA included 27 cases of chondrosarcoma. Some core tissues were removed from the slide during staining for immunohistochemistry (IHC); thus, the presented results only included the samples that remained on the slide and could be graded.

### Cell culture and reagents

HOS, KHOS, 143B, MNNG, U2OS, SAOS-2, MG63, and NIH3T3 cells were obtained from American Type Culture Collection (ATCC). The KHOS cell line used for in vivo experiments was recently authenticated in Beijing Microread Genetics Co., Ltd. by STR analysis and was passaged for less than 3 months after resuscitation. HOS, KHOS, and U2OS cells were cultured in RPMI 1640 medium (HyClone). 143B, MNNG, SAOS-2, MG63, and NIH3T3 cells were maintained in DMEM (HyClone). Cell culture media were supplemented with 10% fetal bovine serum (Gibco) and 1% penicillin/streptomycin (Invitrogen). All cell lines were cultured at 37 °C with 5% CO_2_.

### Western blot

Western blotting was performed as previously described [28]. Briefly, equal amounts of protein were collected from various cell lysates, loaded onto 15% SDS-PAGE gels, resolved using a NuPAGE system (Invitrogen), and transferred onto PVDF membranes. After blocking in non-fat milk for 1 h, the membranes were incubated with corresponding primary antibodies overnight at 4 °C. The bands were probed with the western blot detection system (Bio-Rad, Hercules, CA, USA). Antibodies against PD-L1 (sc-50298) and GAPDH (sc-25778) were purchased from Santa Cruz Biotechnology. Anti-PDL2 (ab187662) was purchased from Abcam.

### Flow cytometry

All osteosarcoma cell lines were analyzed for PD-L1 and PD-L2 expression by flow cytometry. The cells were prepared and incubated with the primary antibody for 30 min at 4 °C and then washed with phosphate-buffered saline (PBS) according to the manufacturer’s instructions. After washing, cells were assayed using an Accuri C6 flow cytometer (BD Biosciences, San Diego, CA, USA). Fluorescent antibodies, including PE-PDL1 (12-5983), APC-PDL2 (17-5888) and the corresponding isotype controls (17-4714 and 12-4714), were purchased from eBioscience. The single-cell suspensions isolated from the mouse tumors were similarly examined for human lymphocyte infiltration by flow cytometry, and the fluorescent antibodies included APC-mouse CD45 (BioLegend, cat#103112), PE-human CD45, PerCP-human CD3, FITC-human CD4, and PE-human CD8a (cat#555483, 347344, 561005, and 340046; BD Pharmingen).

### Immunohistochemistry and immunofluorescence assay

Paraffin sections were incubated with the corresponding antibodies and stained with nonimmune serum in PBS instead of the primary antibody as the negative control. Based on the average percentage of positive cells calculated from at least 10 representative fields (× 400 magnification), positive staining was defined as a positive cell percentage ≥ 10%. Staining intensity was classified as follows: 0, no staining or staining in < 10% of tumor cells; 1+, weak to moderate staining in 10 to 20% of tumor cells; 2+, strong staining in 10 to 20% of tumor cells or weak staining in 20 to 50% of tumor cells; 3+, moderate to strong staining in 20 to 50% of tumor cells or staining in 50% of tumor cells. More than 10 representative areas (× 400 magnification) were calculated for the tumor-infiltrating lymphocyte analysis. The immunostaining assessment was conducted by two independent pathologists without any previous knowledge of the clinical characteristics and outcomes. Antibodies for IHC against PD-L1 (M442) and PD-1 (M569) were purchased from Spring Bioscience. Anti-PD-L2 (82723) was purchased from Cell Signaling Technology, and anti-CD4 (19068-1-AP), anti-CD8a (17335-1-AP), anti-granzyme B (13588-1-AP), and anti-interferon gamma (15365-1-AP) were purchased from Proteintech Group Inc.

For immunofluorescence assay of colocalization of PD-L1 and PD-1 or PD-L2 and PD-1, paraffin sections were incubated with anti-PD-L1 and anti-PD-1 or anti-PD-L2 and anti-PD-1 antibody overnight at 4 °C, then washed three times with PBS and incubated with Alexa Flour 594-conjugated goat anti-mouse IgG and Alexa Flour 488-conjugated goat anti-rabbit IgG for 1 h at room temperature. The sections were viewed using confocal microscopy (FV10i, Olympus, Tokyo, Japan).

### Quantitative RT-PCR

Total RNA was isolated using Trizol (Invitrogen), and cDNAs were synthesized with purified RNA and OligdT primers using SuperScript III First-Strand Synthesis SuperMix (Invitrogen). Real-time quantitative PCR was performed using the SYBR-Green PCR Master Mix (Applied Biosystems, Foster City, CA, USA) on Bio-Rad CFX96 (Applied Biosystems, CA, USA). Relative transcript expression was normalized to GAPDH. All protocols were conducted as per the manufacturer’s instructions.

The primer sequences were as follows: PD-1 forward 5′-AAGCTTATGTGGGTCCGGC-3 and PD-1 reverse 5′-GGATCCTCAAAGAGGCC-3′; PD-L1 forward 5′-ACGCATTTACTGTCACGGTTCC-3′ and PD-L1 reverse 5′-CGATGGGGTTCCGGCTTCAG-3′; PD-L2 forward 5′-AAAGAGCCACTTTGCTGGAG-3′ and PD-L2 reverse 5′-GAGGACGTAGTAACGAAAGT-3′; GAPDH forward 5′-GCACCGTCAAGGCTGAGAAC-3′ and GAPDH reverse 5′-ATGGTGGTGAAGACGCCAGT-3′.

### Datamining and bioinformatic analyses

The osteosarcoma dataset from the Gene Expression Omnibus (GEO) [29] (accession no. GSE21257 [30]) was used for the datamining and bioinformatic analyses in this study. Clustering and heat map visualization was performed using the MeV software, version 4.9. Gene set enrichment analysis [31] (GSEA) was employed to demonstrate the association between our genes of interest and defined gene sets. The gene annotation network analysis was performed using the GATHER [32], BINGO [33], and REVIGO [34] software.

### Establishment of the human PBMC-engrafted mouse model

This study was approved by the Institutional Review Board of Peking University People’s Hospital. Blood samples were collected from donors with written informed consent. NPG mice (NOD prkdc^scid^Il2rg^null^) were purchased from Beijing Vitalstar Biotech. Co. Ltd. The two- to threefold diluted blood samples were subjected to centrifugation on a lymphocyte separation medium (Tianjin Haoyang Biological Manufacture Co. Ltd.) at a density of 1.077 g/ml, and the nucleated cell layer between the plasma and separation medium was gathered. After two washes with RPMI 1640 medium, the PBMC pellets were suspended in RPMI 1640 medium at a density of 5 × 10^7^ cells/ml. Then, 1 × 10^7^ cells were injected via tail vein for each NPG mouse. Mice were housed in an SPF facility and accessed food and water ad libitum. The PBMC-transplanted mice were bled retro-orbitally every week, and the human CD45-positive cell rate in the mouse peripheral blood was analyzed by flow cytometry. The mice with more than 25% human CD45-positive cells in their blood were considered successful human PBMC-engrafted mouse models (see Additional files 1 and 2) and subjected to tumor cell inoculation.

### Generation of xenografts

To evaluate the effect of the nivolumab treatment on primary tumor growth and spontaneous metastasis, 5 × 10^6^ KHOS cells were injected subcutaneously into the right flanks of the humanized mice. On day 5 after the injection, the mice were randomly divided into two groups (*n* = 5 per group) and injected intraperitoneally with sterile saline or nivolumab at a dose of 10 mg/kg every 5 days for a total of five injections. The volume of the xenograft was measured every 5 days (tumor volume = (length × width^2^)/2). The mice were sacrificed after 30 days. At the termination of the study, the lungs were processed for routine hematoxylin-eosin (H&E) staining, and the number of metastatic nodules in the lung was determined. The tumors collected from the mice were chopped into small pieces and digested with 1 mg/ml collagenase type IV (Worthington, NJ) solution for 30 min. The dissociated tissues were processed through a 70-μm strainer, and the single-cell suspensions were subjected to flow cytometric analysis for human CD4+ and CD8+ lymphocyte infiltration.

### Statistical analysis

All statistical analyses were performed using the SPSS v.21.0 software (SPSS, Chicago, IL, USA) and the GraphPad Prism software. For survival analysis, overall survival (OS) was defined as the time interval between the confirmed diagnosis and death or last follow-up. Survival analysis was per the Kaplan-Meier method with the log-rank test. The association between the expression levels of the PD-1 axis effectors and the clinicopathological variables, along with the relationships among the expression levels of the PD-1 axis effectors, were assessed by using the chi-square analysis. Statistical evaluations were performed using Student’s *t* tests. Data are expressed as the mean ± S.D. In all statistical analyses, a *P* value < 0.05 was considered statistically significant in the two-sided test.

## Results

### PD-L1, PD-L2, and PD-1 expression patterns and clinicopathological features in musculoskeletal tumors

**Fig. 1:**
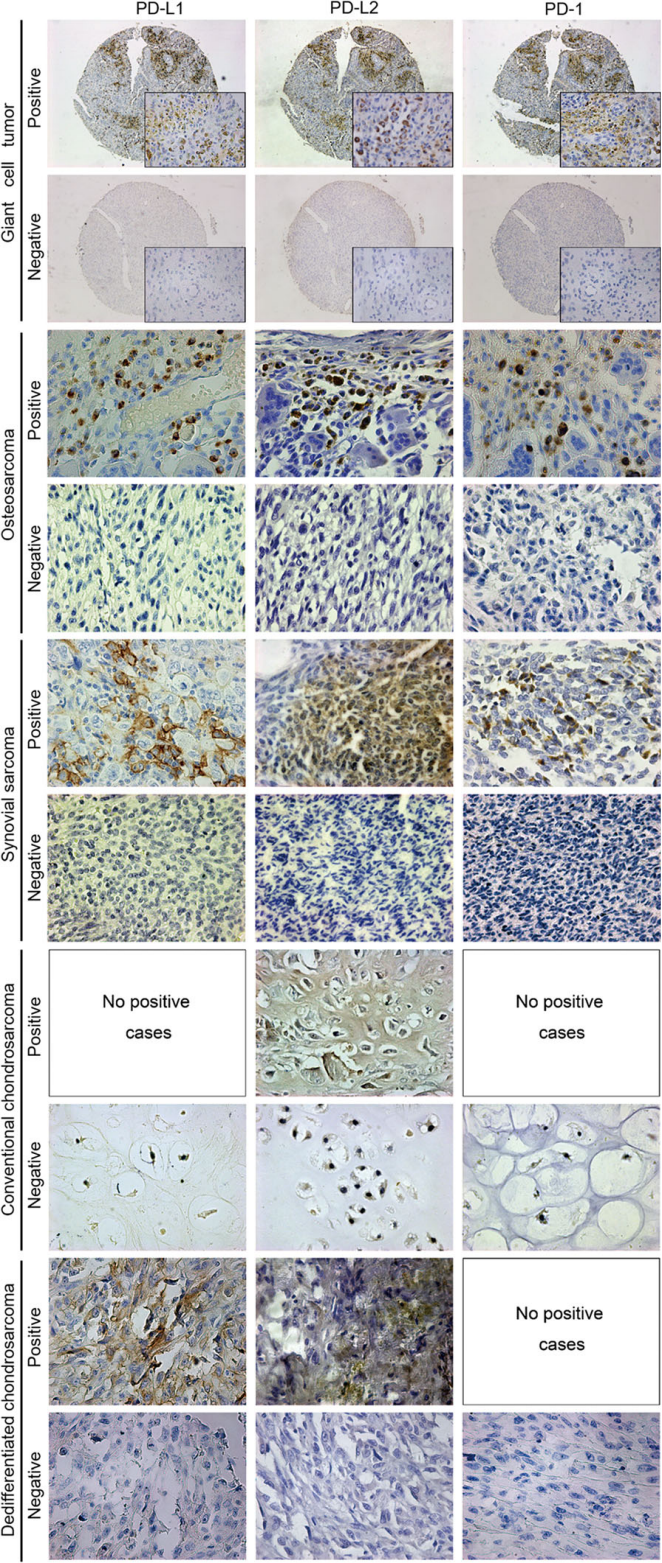
IHC staining for PD-L1, PD-L2, and PD-1 expression in the TMA samples. PD-L1, PD-L2, and PD-1 exhibited a membranous expression accompanied by cytoplasmic expression. Representative images for every histological type are shown (The background image was magnified at × 50, while the insert image was magnified at × 400 in GCT. The other histological types were magnified at × 400)

PD-L1, PD-L2, and PD-1 expression patterns were examined in a musculoskeletal tumor TMA (234 cases), including osteosarcoma (62 cases), chondrosarcoma (31 cases), synovial sarcoma (127 cases), and GCT (14 cases), using IHC. Representative positively and negatively stained images for each pathological type are shown in Fig. 1.

As shown in Table 1, PD-L1, PD-L2, and PD-1 positivity was detected in 55 cases (23.5%), 66 cases (28.2%), and 49 cases (20.9%), respectively, of musculoskeletal tumors. In particular, the positivity rates of PD-L1, PD-L2, and PD-1 in osteosarcoma were 35.5% (22/62), 41.9% (26/62), and 27.4% (17/62), respectively. Similarly, the positivity rates of PD-L1, PD-L2, and PD-1 in synovial sarcoma were 15.7% (20/127), 20.5% (26/127), and 18.9% (24/127), respectively. PD-L1 was not expressed in conventional chondrosarcoma (0/27), but it was detected in dedifferentiated chondrosarcoma (DDCS) (3/4). The positivity rates of PD-L2 and PD-1 were 22.2% (6/27) and 0% (0/27), respectively, in conventional chondrosarcoma and 25.0% (1/4) and 0% (0/4), respectively, in DDCS. Interestingly, high levels of PD-L1 (71.4%, 10/14), PD-L2 (50.0%, 7/14), and PD-1 (57.1%, 8/14) expressions were detected in GCT for the first time. Furthermore, double immunofluorescence staining indicated the colocalization of PD-L1/PD-1 and PD-L2/PD-1 in osteosarcoma (Additional file 2).

**Table 1:** Expression of PD-L1, PD-L2, and PD-1 in musculoskeletal tumors

Tumor	*N*	PD-L1 *N* (%)				PD-L2 *N* (%)						PD-1 *N* (%)				
		0	1+	2+	3+	Positive	0	1+	2+	3+	Positive	0	1+	2+	3+	Positive
Musculoskeletal tumor	234	179	35	10	10	55 (23.5%)	168	49	8	9	66 (28.2%)	185	37	5	7	49 (20.9%)
Giant cell tumor	14	4	1	2	7	10 (71.4%)	7	1	1	5	7 (50.0%)	6	1	2	5	8 (57.1%)
Osteosarcoma	62	40	17	3	2	22 (35.5%)	36	21	3	2	26 (41.9%)	45	15	1	1	17 (27.4%)
Synovial sarcoma	127	107	16	3	1	20 (15.7%)	101	22	2	2	26 (20.5%)	103	21	2	1	24 (18.9%)
Chondrosarcoma	31	28	1	2	0	3 (9.7%)	24	5	2	0	7 (22.6%)	31	0	0	0	0
Conventional chondrosarcoma	27	27	0	0	0	0	21	4	2	0	6 (22.2%)	27	0	0	0	0
Dedifferentiated chondrosarcoma	4	1	1	2	0	3 (75.0%)	3	1	0	0	1 (25.0%)	4	0	0	0	0

Abbreviations: *PD-L1* programmed death ligand-1, *PD-L2* programmed death ligand-2, *PD-1* programmed death-1

As shown in Table 2, only PD-L2 expression was significantly associated with PD-1 expression in the sarcomas (*P* = 0.036 for PD-L2 versus PD-1). In particular, similar results were observed in chondrosarcoma (*P* = 0.016 for PD-L2 versus PD-1), while no association was observed in synovial sarcoma and osteosarcoma. However, when PD-L1 and PD-L2 expression was taken together for analysis with PD-1 expression, the expression levels of the two PD-1 ligands were significantly correlated with PD-1 expression in the sarcomas (*P* = 0.000) and in osteosarcoma (*P* = 0.002), synovial sarcoma (*P* = 0.017) and chondrosarcoma (*P* = 0.002).

**Table 2:** Association of PD-L1, PD-L2, and PD-1 expressions in sarcomas

Tumor	Association of PD-1 axis expressions (*P* value)			
	PD-L1 vs PD-L2	PD-L1 vs PD-1	PD-L2 vs PD-1	Combined PD-Ls vs PD-1
Sarcomas	0.104	0.694	0.036	0.000
Synovial sarcoma	0.405	0.617	0.868	0.017
Osteosarcoma	0.481	0.359	0.093	0.002
Chondrosarcoma	0.344	0.250	0.016	0.002

Abbreviations: *PD-L1* programmed death ligand-1, *PD-L2* programmed death ligand-2, *PD-1* programmed death-1

The PD-L1, PD-L2, and PD-1 expression levels were significantly different according to the histologic type (*P* = 0.000 for PD-L1; *P* = 0.004 for PD-L2; *P* = 0.000 for PD-1), while no significant difference regarding age and sex was evident for the sarcomas. Specifically, the PD-L1, PD-L2, and PD-1 expression levels were not significantly different among primary, recurrent, and metastatic osteosarcoma. In synovial sarcoma, PD-L1 expression was significantly different according to clinical stage (*P* = 0.011), while no difference was evident for PD-L2 (*P* = 0.912) and PD-1 (*P* = 0.103) (Table 3).

**Table 3:** Relationship between PD-1 axis expression and clinicopathological features in musculoskeletal tumors

Variables		*N* %	PD-L1 expression			PD-L2 expression			PD-1 expression		
			+	−	*P*	+	−	*P*	+	−	*P*
Sex	Male	129	32	97	0.603	36	93	0.911	30	99	0.335
	Female	105	23	82		30	75		19	86	
Age	≤ 20	47	15	32	0.293	19	28	0.144	9	38	0.165
	≤ 33	65	13	52		17	48		18	47	
	≤ 50	75	14	61		16	59		10	65	
	> 50	47	13	34		14	33		12	35	
Histologic type of musculoskeletal tumor	Synovial sarcoma	127	20	107	0.000	26	101	0.004	24	103	0.000
	Osteosarcoma	62	22	40		26	36		17	45	
	Chondrosarcoma	31	3	28		7	24		0	31	
	Giant cell tumor	14	10	4		7	7		8	6	
State of osteosarcoma*	Primary osteosarcoma	45	15	30	0.225	18	27	0.878	10	36	0.087
	Recurrent osteosarcoma	17	7	10		8	9		7	9	
	Metastatic osteosarcoma	5	0	5		2	3		0	5	
Clinical stage of synovial sarcoma	G1	16	0	16	0.011	3	13	0.912	3	13	0.103
	G2-3	99	15	84		20	79		16	83	
	G4	12	5	7		3	9		5	7	

Abbreviations: *PD-L1* programmed death ligand-1, *PD-L2* programmed death ligand-2, *PD-1* programmed death-1

*5 paired samples included on the array (62 osteosarcoma patients with 67 samples)

The PD-L1, PD-L2, and PD-1 RT-PCR assay was performed on total RNA isolated from 12 human osteosarcoma and 12 normal bone tissue samples. Among them, 12 osteosarcoma samples were from IHC cohort. Compared to normal bone tissue, the PD-L1, PD-L2, and PD-1 mRNA expressions were significantly higher in osteosarcoma (Additional file 3).

### Survival analyses according to PD-L1, PD-L2, and PD-1 expression in osteosarcoma

**Fig. 2:**
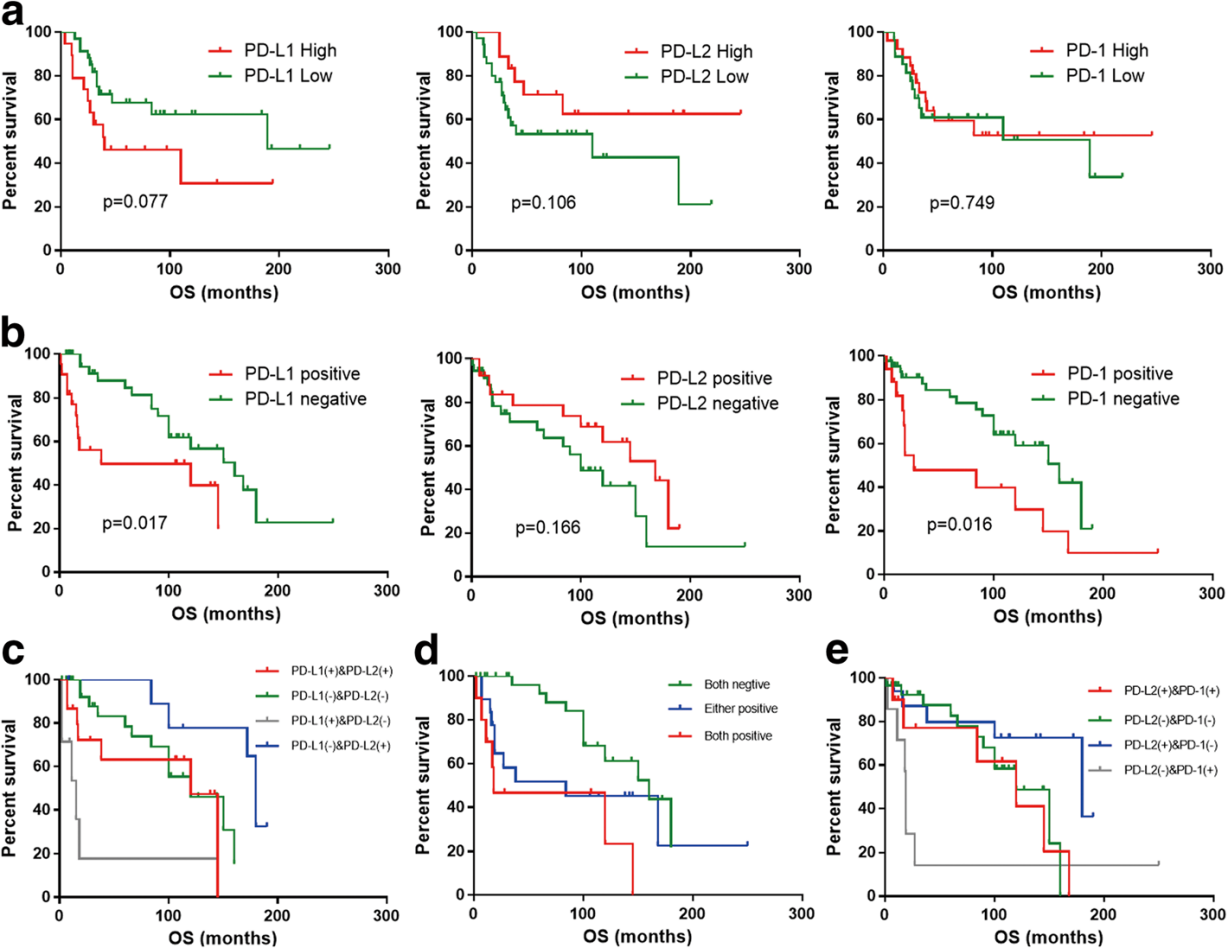
Survival analyses according to the PD-L1, PD-L2, and PD-1 expression levels in the GEO and IHC cohorts. **a** OS according to PD-L1, PD-L2, and PD-1 in the GEO cohort. **b** OS according to PD-L1, PD-L2, and PD-1 in the IHC cohort. **c** Subgroup survival analyses according to PD-L1 and PD-L2 expression in the IHC cohort. **d** Subgroup survival analyses according to PD-L1 and PD-1 expression in the IHC cohort. **e** Subgroup survival analyses according to PD-L2 and PD-1 expression in the IHC cohort

To evaluate the relationship between the expression patterns of the PD-1 axis and survival in osteosarcoma, we first analyzed OS in the GSE21257 dataset (53 cases) and found that PD-L1 expression had a negative correlation tendency with OS (*P* = 0.077), while PD-1 expression had no correlation with OS (*P* = 0.749). PD-L2 expression had a positive correlation trend with OS (*P* = 0.106) (Fig. 2a).

Furthermore, we analyzed the survival curve of 62 osteosarcoma TMA patients, and high expression of PD-L1 significantly predicted a short OS time (*P* = 0.017). Similarly, PD-1 expression was also negatively associated with OS (*P* = 0.016). Unlike PD-L1 and PD-1, the PD-L2-positive group had a longer OS time than the PD-L2-negative group, although the *P* value did not achieve statistical significance (*P* = 0.166), which meant that PD-L2 expression had a positive correlation trend with OS in osteosarcoma (Fig. 2b).

Based on the PD-L1 and PD-L2 expression levels, the osteosarcoma TMA patients were divided into four subgroups. As shown in Fig. 2c, the PD-L2-positive subgroup had a better OS than the PD-L2-negative subgroup (*P* = 0.036) in PD-L1-positive group, and the PD-L2-positive subgroup exhibited a borderline positive correlation trend with OS (*P* = 0.076) in the PD-L1-negative group. In contrast, PD-L1 expression indicated a negative correlation tendency with OS (*P* = 0.051) in the PD-L2-positive group. Similarly, in the PD-L2-negative group, PD-L1 expression predicted a negative correlation with OS (*P* = 0.000).

Considering the independent negative prognostic roles of PD-L1 and PD-1, we further divided the patients into three groups on the basis of PD-L1 and PD-1 expression to correlate the combined expression of PD-L1 and PD-1 with OS as follows: (I) positivity for both PD-L1 and PD-1 (*n* = 10), (II) positivity for either PD-L1 or PD-1 (*n* = 19), and (III) negativity for both PD-L1 and PD-1 (*n* = 33). The doubly positive group had a distinctly worse OS than the doubly negative group (*P* = 0.001), whereas the statistical significance between the doubly negative group and the singly positive group was borderline (*P* = 0.085). The doubly positive group tended to have a worse OS than the singly positive group, though statistical significance was not achieved (median survival, 160 versus 84 months, *P* = 0.215) (Fig. 2d).

On the basis of the PD-L2 and PD-1 expression levels, four subgroups were formed for an OS analysis. As shown in Fig. 2e, in the PD-L2-positive group, PD-1 expression was negatively correlated with OS (*P* = 0.043). Similarly, in the PD-L2-negative group, PD-1 expression had a negative correlation with OS (*P* = 0.024). Conversely, in the PD-1-negative group, PD-L2 expression indicated a longer OS time (*P* = 0.084). The most obvious discrepancy in median survival was between the PD-L2(+) and PD-1(−) subgroup and PD-L2(−) and PD-1(+) subgroup (*P* = 0.014). The doubly positive subgroup was not significantly different from the doubly negative subgroup (*P* = 0.864).

Taken together, these survival analyses of the IHC and GEO data reveal that PD-L1 was negatively correlated with prognosis, while PD-1 had a negative correlation tendency with OS. Meanwhile, PD-L2 had a positive correlation trend with OS.

### Potential mechanisms that underlie the PD-1, PD-L1, and PD-L2 associations with prognosis

**Fig. 3:**
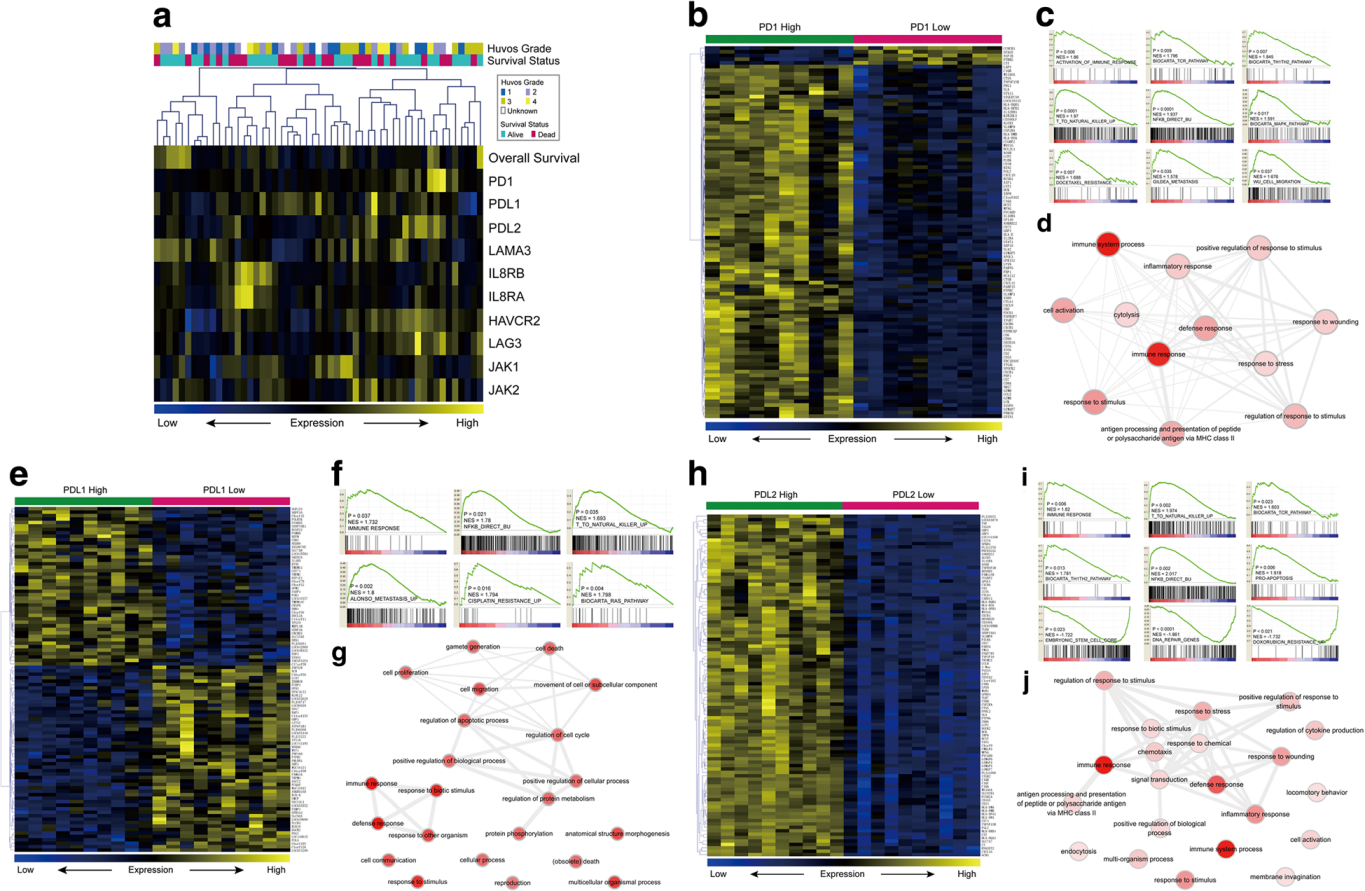
The underlying mechanisms of the PD-1, PD-L1 and PD-L2 associations with prognosis. **a** Cluster analysis and heat map visualization of immune checkpoint-related genes, OS and HUVOS grade. **b** Heat map visualization of the differentially expressed genes between the PD-1 high-expression group and PD-1 low-expression group, **c** GSEA analysis of PD-1 and **d** gene annotation network analysis of the differentially expressed genes. **e** Heat map visualization of the differentially expressed genes between the PD-L1 high-expression group and PD-L1 low-expression group, **f** GSEA analysis of PD-L1 and **g** gene annotation network analysis of the differentially expressed genes. **h** Heat map visualization of the differentially expressed genes between the PD-L2 high-expression group and PD-L2 low-expression group, **i** GSEA analysis of PD-L2 and **j** gene annotation network analysis of the differentially expressed genes

An overview of the expression patterns of immune checkpoint-related genes (including PD-1, PD-L1, and PD-L2) and their associations with OS and HUVOS grade in the 53 osteosarcoma samples is shown in Fig. 3a. To explore the underlying molecular mechanisms of the PD-1, PD-L1, and PD-L2 associations with the clinical features and prognosis, gene expression patterns, function enrichment, and gene annotation network analyses of genes associated with PD-1, PD-L1, or PD-L2 were computed and visualized. Figure 3b shows the heat map visualization of the top 100 differentially expressed genes between the PD-1 high-expression group and the PD-1 low-expression group (top 10 versus bottom 10 samples). Similarly, Fig. 3e, h demonstrates the expression patterns of the PD-L1- and PD-L2-related genes.

The GSEA analysis results show that PD-1 is positively correlated with activation of immune response pathways, docetaxel resistance, and metastasis-related signatures (Fig. 3c). PD-L1 is positively associated with activation of the immune response and RAS pathways, metastasis signatures, and cisplatin resistance (Fig. 3f). PD-L2 is positively correlated with immune pathway activation and pro-apoptosis signatures but negatively correlated with stem cell, DNA repair, and doxorubicin resistance signatures (Fig. 3i).

Gene annotation network analyses were performed on the differentially expressed genes (Fig. 3d: PD-1 high versus PD-1 low; Fig. 3g: PD-L1 high versus PD-L1 low; Fig. 3j: PD-L2 high versus PD-L2 low). These figures show that the immune response and stimulus and wounding responses are common processes that are enriched by the differentially expressed genes.

### PD-L1 and PD-L2 expression in osteosarcoma cell lines

**Fig. 4:**
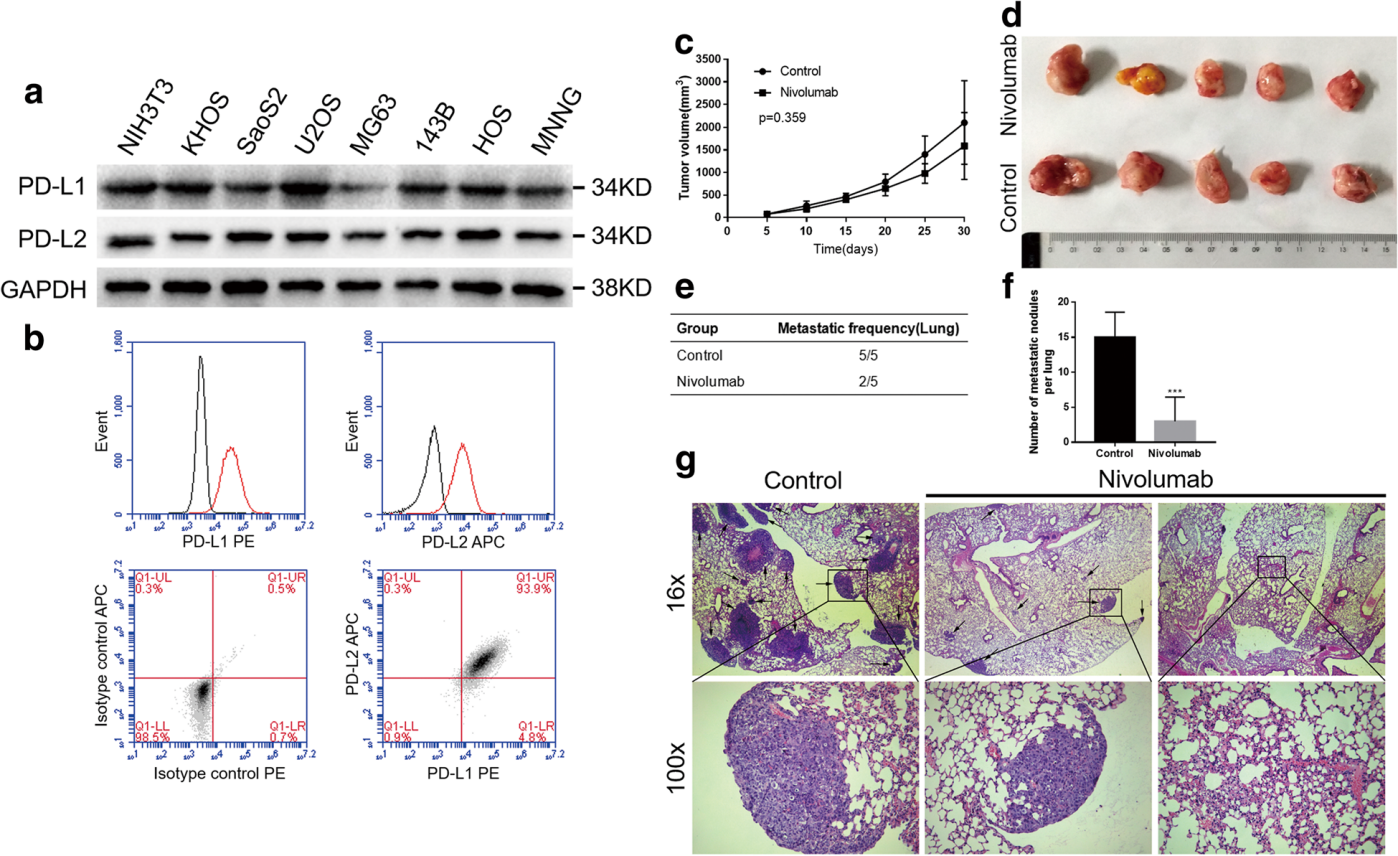
The expressions of PD-L1 and PD-L2 in osteosarcoma cell lines and nivolumab-suppressed osteosarcoma metastasis in vivo. **a** Western blotting analyses of PD-L1 and PD-L2 expression. NIH3T3 was used as the positive control. **b** Representative membranous expression of PD-L1 and PD-L2 in the KHOS cell line (red) compared with that of the isotype control (black) from the flow cytometry analysis. **c** The tumor growth curve of the nivolumab-treated and control groups. **d** Representative images of the primary tumors. **e** Metastatic frequency of the nivolumab-treated and control groups. **f** The number of metastatic nodules in the lungs from the nivolumab-treated and control groups is presented. **g** H&E staining of the lungs from the nivolumab-treated and control groups (magnification at × 16 and × 100). The lung metastases are indicated by the arrows. Data are presented as the mean ± S.D. (*n* = 5) ***P < 0.001 by Student’s *t* test

Western blot and flow cytometric analyses were performed on seven osteosarcoma cell lines for total protein and cell surface protein detection, respectively. With NIH3T3 cells as the positive control, the western blot analysis indicated that the HOS, KHOS, 143B, MNNG, SAOS-2, and U2OS cell lines showed relatively higher levels of PD-L1 protein than the MG63 cell line, while all osteosarcoma cell lines showed high levels of PD-L2 expression (Fig. 4a). The flow cytometric assays revealed that the KHOS cells showed distinct membranous expression patterns of PD-L1 and PD-L2 compared with those of the isotype control and that the other cell lines exhibited differing degrees of expression (Fig. 4b and Additional file 3). The KHOS cell line was chosen for further study in vivo.

### Nivolumab inhibits osteosarcoma metastasis in vivo

On the basis of the negative prognostic roles of PD-1 and PD-L1, we examined whether a PD-1/PD-L1 interaction blockade with nivolumab would affect osteosarcoma growth and metastasis in vivo. Five days after the KHOS cells were injected subcutaneously into the right armpits of humanized mice, the mice were randomly divided into two groups and intraperitoneally administered nivolumab (10 mg/kg bodyweight) or sterile saline every 5 days for a five-injection treatment course.

**Fig. 5:**
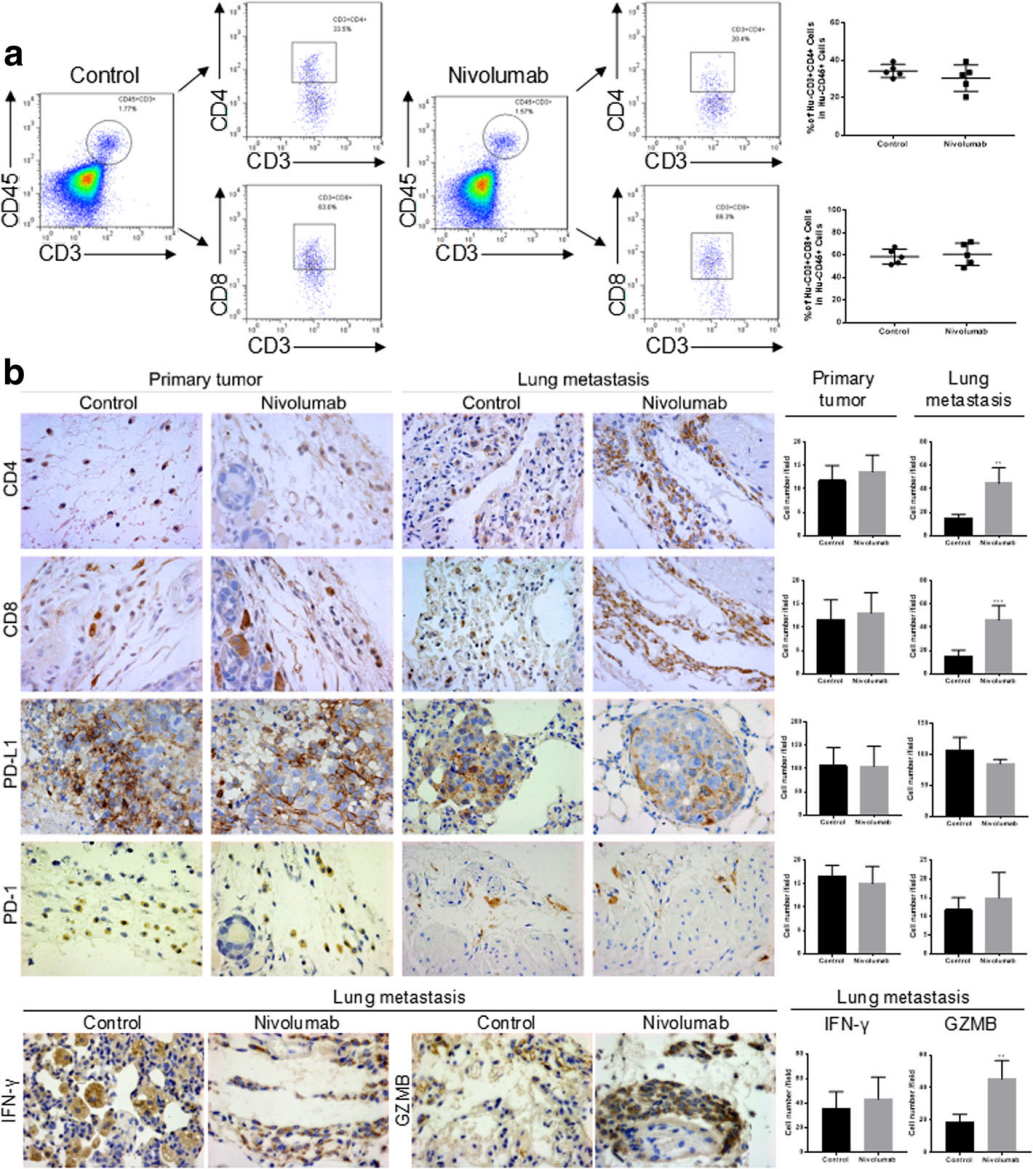
Effect of the nivolumab treatment on TILs. **a** Flow cytometry analyses of TILs in tumors from the nivolumab-treated and control groups. **b** IHC analyses of CD4, CD8, PD-L1, PD-1, GZMB, and IFN-γ in the tumors and lungs of the nivolumab-treated and control groups (magnification at × 400). Data are presented as the mean ± S.D. (*n* = 5) **P < 0.01, ***P < 0.001 by Student’s *t* test

No significant differences were observed in the primary tumor volume and growth rate between the nivolumab-treated group and control group (Fig. 4c–d). This result indicated that nivolumab had no effect on tumor formation in vivo. Lung metastases were found in all five mice (5/5) of the control group, while lung metastatic nodes were found in a portion of the mice (2/5) of the nivolumab-treated group (Fig. 4e). The nivolumab-treated group exhibited significantly fewer lung metastatic nodes than the control group (Fig. 4f). Representative H&E images of the lungs are shown in Fig. 5g.

Overall, nivolumab markedly suppressed the metastatic potential of osteosarcoma but not primary osteosarcoma in vivo.

### Analyses of tumor-infiltrating lymphocytes in tumors and lung metastases in humanized mice

The flow cytometry results showed that the primary tumors in the humanized PBMC-NPG mice were infiltrated with human CD4+ and CD8+ lymphocytes, but both groups displayed similar proportions of these T cells regardless of treatment (Fig. 5a).

The IHC results indicated that CD4+ and CD8+ lymphocytes were more frequently observed in the lungs of the nivolumab-treated group than in the lungs of the control group, while CD4+ and CD8+ lymphocytes showed no statistically significant differences in the primary tumors from both groups, which was consistent with the flow cytometry results. PD-L1 and PD-1 were also detected by IHC in tumors and lung metastases, and no differences were observed between the two groups (Fig. 5b). Additionally, the IHC assay for granzyme B (GZMB) and IFN-γ indicated that GZMB exhibited a higher expression in the lungs of the nivolumab-treated group than that of the control group, while IFN-γ showed no statistically significant differences between these two groups.

These data reveal that nivolumab enhances tumor lymphocyte infiltration and the cytolytic activity of CD8 lymphocytes in lung metastases, which may be the mechanism by which nivolumab inhibits lung metastasis.

## Discussion

Known as the B7 family members, PD-L1 and PD-L2 both provide negative costimulatory signals during antigen-specific T cell activation by binding to the PD-1 receptor. Therefore, these three effectors play important roles in forming the tumor immunosuppressive microenvironment. Several studies have shown that inhibition of the PD-1 axis restores and enhances the immune response in vitro and in vivo [35, 36]. Meanwhile, multiple clinical trials have suggested that blocking the interaction between PD-1 and PD-L1 can effectively inhibit tumor progression and improve patient prognosis [5]. It is important to note that patients with high levels of PD-L1 expression have higher response rates to PD-1 antibody immunotherapy than patients with low PD-L1 expression [37], but some patients with little or no expression of PD-L1 also respond to immunotherapy. This phenomenon may be due to PD-L2 expression in this cohort of patients; the PD-1 antibody may block the interaction between PD-L2 and PD-1. Therefore, many scholars have begun regarding PD-L1, PD-L2, and PD-1 as a whole and systematically studying the clinical value of these effectors as biomarkers to predict patient prognosis and evaluate the potential effects of the ICI treatment [13–17].

Few studies have analyzed the clinical significance of the PD-1 axis, especially PD-L2, in sarcomas. In our study, we examined the expression levels of PD-L1, PD-L2, and PD-1 in multiple sarcomas, as indicated in the “Results” section. The existing studies indicate that PD-L1 mRNA expression is detectable in osteosarcoma and exhibits a negative borderline trend with OS [20]. Moreover, metastatic, but not primary, osteosarcoma tumors express PD-L1 and PD-1 [21, 22], while a recent study has shown that PD-L1 is detectable in both primary and metastatic osteosarcomas, with no significant differences between them [23]. In our study, no significant differences were observed in the expression levels of PD-L1, PD-L2, and PD-1 among the primary, recurrent, and metastatic osteosarcomas, which may be due to the relatively few metastatic osteosarcoma patients (5 patients). The positivity rates of PD-L1 and PD-1 in synovial sarcoma were similar to those in previous studies [24, 25], while PD-L2 expression was detected in 26 cases (20.5%) for the first time. Furthermore, PD-L1 expression was significantly different according to clinical stage in synovial sarcoma, while PD-1 expression had a borderline difference. Similarly, a recent study reported that PD-L1 and PD-1 expression in tumor-invasive margins was significantly higher in metastatic synovial sarcoma than in primary synovial sarcoma and that PD-1 expression in the tumor-invasive margin was negatively associated with progression-free survival [27]. We also found that PD-L1 was not expressed in conventional chondrosarcoma, while PD-L1 was detectable in DDCS, which was consistent with a previous study [26]; this PD-L1 expression difference is possibly because the dedifferentiated component in DDCS may be osteosarcoma, which can express PD-L1. A previous study highlighted PD-L1 expression in some sarcoma patients with a history of GCT [25], so we further investigated the expression levels of PD-L1, PD-L2, and PD-1 in GCT. As mentioned above, we detected high levels of PD-L1, PD-L2, and PD-1 expressions in GCT for the first time. In our study, a significant association was observed between expression of the combined PD-1 ligands and expression of PD-1 not only in sarcomas as a whole but also in osteosarcoma, synovial sarcoma, and chondrosarcoma, which indicates that PD-L1 and PD-L2 should be regarded as a whole for systematic investigations. Moreover, the expression patterns of PD-1 axis are varying in different tumors: the positive rates of PD-L1 and PD-L2 in lung adenocarcinoma are about 50% [14]; PD-L1 and PD-L2 expressions are observed in 9.4 and 49.6% of renal cell carcinoma patients, respectively [15]; in metastatic melanomas, positive rates of PD-L1 and PD-1 are 49 and 25%, respectively [17]; high expressions of PD-L1 and PD-1 are detected in 38.4 and 50% of colorectal cancer patients, respectively [19]. Variety of PD-1 axis expression in different tumors may lead to their different responses to immunotherapy. Previous study also indicated a statistically significant correlation between PD-L1 and PD-L2 in lung cancer [18] and showed that PD-1, PD-L1, and PD-L2 have different prognostic roles in various tumors [8–19].

Because the PD-1 axis effectors are differentially expressed in various sarcomas, the immunotherapeutic efficiency may vary widely due to the pathological type of the sarcoma, so further investigation of each sarcoma is urgently needed. Based on our findings, osteosarcoma, which is the most common primary malignant bone tumor with a high mortality and disability rate, exhibits relatively high expression levels of PD-1 axis effectors. Furthermore, the IHC experiments and data mining both indicate that PD-L1 was negatively correlated with prognosis, while PD-1 had a negative correlation tendency with OS. Meanwhile, PD-L2 had a positive correlation trend with OS.

To investigate the potential mechanisms that underlie the PD-1, PD-L1, and PD-L2 associations with prognosis, mRNA expression of 10 immune checkpoint-related genes in the osteosarcoma samples were clustered and visualized through datamining and bioinformatic analyses. The expression patterns, GSEA, and gene annotation network analysis of the genes associated with PD-1, PD-L1, or PD-L2 have also been presented. In addition to immune suppression, our results indicate that PD-1 may be correlated with docetaxel resistance and activation of MAPK and metastasis-related pathways. The PD-L1-associated poor prognosis may due to immune suppression, cisplatin resistance, and metastasis-related pathway activation, whereas PD-L2 may slow osteosarcoma progression by repressing DNA repair, stem cell-related pathways, and doxorubicin resistance.

Based on the negative prognostic role of PD-1/PD-L1 and the immune response associated with the PD-1 axis in the datamining analysis, we investigated whether blockade of the PD-1 axis could generate an antitumor effect in osteosarcoma. In our study, we revealed that treatment with nivolumab resulted in effective control of pulmonary metastasis in an osteosarcoma model in humanized mice, while no obvious effect was evident on localized osteosarcoma. Moreover, the tumor-infiltrating lymphocyte (TIL) investigation indicated that nivolumab increased CD4+ and CD8+ lymphocytes in the lung but not in the primary lesion. Furthermore, nivolumab enhances the cytolytic activity of CD8 lymphocytes in the lung. The limitation of this animal model was that the mature human lymphocytes gradually initiated severe graft-versus-host disease in the mouse due to the PBMC injection, which resulted in a relatively short survival time. Therefore, we could not determine whether nivolumab could control tumor growth over the long term, even though the tumor growth rate of the nivolumab-treated group had begun to slow down in our study.

As we know, ICI inhibits tumor development by restoring the functions of T cells to kill the tumor cells, and the quantity of TILs plays an important role in the immunotherapy effect. In our study, nivolumab inhibited osteosarcoma metastasis by increasing the number of lymphocytes in the lung but have been ineffective towards primary osteosarcoma. Interestingly, in recent studies [38, 39], patients with PD-L1/PD-1 monoclonal antibody therapy showed a pattern of rapid disease progression. One reason suggested by the authors was that the PD-1/PD-L1 axis mediated inherent functions in the tumor cells and that the PD-1/PD-L1 blockade may have affected the tumor cell-intrinsic signaling network and subsequently enhanced tumor growth or progression. This indicates that the treatment effect of ICI may be associated with tumor cell-intrinsic signaling of PD-L1 and PD-L2. Several studies have found that PD-L1 and PD-L2 are associated with multiple cellular biological behaviors, such as the epithelial-mesenchymal transition (EMT), proliferation, and autophagy [40–43]. At present, no relevant studies have been conducted to address the tumor cell-intrinsic effects of PD-L1 or PD-L2 in osteosarcoma; thus, further investigations should be undertaken to improve the treatment effect of ICI.

## Conclusions

In summary, this study is the first to systematically investigate the expression patterns of PD-1/PD-L1/PD-L2 in osteosarcoma, chondrosarcoma, synovial sarcoma, and GCT. The diversity of the expression levels of PD-1, PD-L1, and PD-L2 may indicate the underlying basis for the different immunotherapy outcomes. The bioinformatic analysis and our TMA results revealed that PD-L1 was negatively correlated with prognosis, while PD-1 had a negative correlation tendency with OS. Meanwhile, PD-L2 had a positive correlation trend with OS in osteosarcoma. The PD-1- and PD-L1-associated poor prognosis in osteosarcoma may due to immune suppression, chemotherapy resistance, and metastasis-related pathways. Our in vivo experiments demonstrated that nivolumab inhibited lung metastasis of osteosarcoma rather than primary tumor growth by increasing the numbers of CD4+ and CD8+ lymphocytes as well as cytolytic activity of CD8 lymphocytes in the lung. Further experiments are needed to confirm the mechanism involved and whether the PD-1 axis is a potential and promising immunotherapeutic target for other sarcomas.

## Additional files

Additional file 1: Table S1. The summary of examination for human CD45 positive cells in humanized mice by flow cytometry. (DOCX 13 kb)

Additional file 2: Figure S1. Representative images of the assessment of the human CD45 positivity cell rate in humanized mice and immunofluorescence assay for PD-L1/PD-1 and PD-L2/PD-1 in osteosarcoma. (DOCX 1962 kb)

Additional file 3: Figure S2. PD-L1, PD-L2 and PD-1 expressions in osteosarcoma. (DOCX 1426 kb)

## Abbreviations

DDCS: Dedifferentiated chondrosarcoma
EMT: Epithelial-mesenchymal transition
GCT: Giant cell tumor
ICI: Immune checkpoint inhibitor
OS: Overall survival
PBMC: Peripheral blood mononuclear cell
PD-1: Programmed death-1
PD-L1: Programmed death ligand-1
PD-L2: Programmed death ligand-2
TIL: Tumor-infiltrating lymphocyte
TMA: Tissue microarray
