# Correction to: PD-1 axis expression in musculoskeletal tumors and antitumor effect of nivolumab in osteosarcoma model of humanized mouse

**DOI:** 10.1186/s13045-018-0580-x

**Published:** 2018-03-12

**Authors:** Bingxin Zheng, Tingting Ren, Yi Huang, Kunkun Sun, Shidong Wang, Xing Bao, Kuisheng Liu, Wei Guo

**Affiliations:** 1 Musculoskeletal Tumor Center, Peking University People’s Hospital, No. 11 Xizhimen South Street, Beijing, 100044 People’s Republic of China; 2 Beijing Key Laboratory of Musculoskeletal Tumor, Beijing, People’s Republic of China; 3 Department of Pathology, Peking University People’s Hospital, Beijing, People’s Republic of China

## Erratum

The original article [1] contained an error in Table 1 whereby the ‘Positive’ column in the ‘PD-L1’ Tumor type group of columns was mistakenly included at the beginning of the ‘PD-L2’ Tumor type group of columns.

This error has now been corrected. Furthermore, this error was not the fault of the authors, and was instead mistakenly carried forward by the production team that handled this article.

**Table 1:** Expression of PD-L1, PD-L2, and PD-1 in musculoskeletal tumors

Tumor	*N*	PD-L1 *N* (%)					PD-L2 *N* (%)					PD-1 *N* (%)				
		0	1+	2+	3+	Positive	0	1+	2+	3+	Positive	0	1+	2+	3+	Positive
Musculoskeletal tumor	234	179	35	10	10	55 (23.5%)	168	49	8	9	66 (28.2%)	185	37	5	7	49 (20.9%)
Giant cell tumor	14	4	1	2	7	10 (71.4%)	7	1	1	5	7 (50.0%)	6	1	2	5	8 (57.1%)
Osteosarcoma	62	40	17	3	2	22 (35.5%)	36	21	3	2	26 (41.9%)	45	15	1	1	17 (27.4%)
Synovial sarcoma	127	107	16	3	1	20 (15.7%)	101	22	2	2	26 (20.5%)	103	21	2	1	24 (18.9%)
Chondrosarcoma	31	28	1	2	0	3 (9.7%)	24	5	2	0	7 (22.6%)	31	0	0	0	0
Conventional chondrosarcoma	27	27	0	0	0	0	21	4	2	0	6 (22.2%)	27	0	0	0	0
Dedifferentiated chondrosarcoma	4	1	1	2	0	3 (75.0%)	3	1	0	0	1 (25.0%)	4	0	0	0	0
