# Supplementary material for: PD-1 axis expression in musculoskeletal tumors and antitumor effect of nivolumab in osteosarcoma model of humanized mouse
Source: J Hematol Oncol. 2018 Feb 6;11:16. doi: 10.1186/s13045-018-0560-1 (PMC5801803; doi:10.1186/s13045-018-0560-1)
Supplement: Supplementary file 1 — The summary of examination for human CD45 positive cells in humanized mice by flow cytometry. (DOCX 13 kb) [file 13045_2018_560_MOESM1_ESM.docx]

Table S1: The summary of examination for human CD45 positive cells in humanized mice by flow cytometry

| Cat.No. | human CD45 % of all | mouse CD45 % of all | hCD45/(hCD45+mCD45) | hCD3/ hCD45 |
| --- | --- | --- | --- | --- |
| PBMC-1 | 23.9 | 17.9 | 57.2 | 86.2 |
| PBMC-2 | 20.1 | 30.5 | 39.7 | 89.3 |
| PBMC-3 | 4.0 | 2.72 | 59.5 | 87.4 |
| PBMC-4 | 2.24 | 3.87 | 36.7 | 82.8 |
| PBMC-5 | 2.14 | 1.17 | 64.7 | 82.6 |
| PBMC-6 | 13.2 | 10.8 | 55.0 | 76.7 |
| PBMC-7 | 3.08 | 1.19 | 72.1 | 83.8 |
| PBMC-8 | 1.95 | 1.38 | 58.6 | 88.6 |
| PBMC-9 | 2.18 | 1.68 | 56.5 | 76.7 |
| PBMC-10 | 8.5 | 8.43 | 50.2 | 84.3 |
| PBMC-11 | 2.13 | 4.66 | 31.4 | 80.1 |
| PBMC-12 | 1.29 | 0.873 | 59.6 | 91.3 |

Abbreviations: PBMC: peripheral blood mononuclear cell.
