# Supplementary material for: PD-1 axis expression in musculoskeletal tumors and antitumor effect of nivolumab in osteosarcoma model of humanized mouse
Source: J Hematol Oncol. 2018 Feb 6;11:16. doi: 10.1186/s13045-018-0560-1 (PMC5801803; doi:10.1186/s13045-018-0560-1)
Supplement: Supplementary file 2 — Representative images of the assessment of the human CD45 positivity cell rate in humanized mice and immunofluorescence assay for PD-L1/PD-1 and PD-L2/PD-1 in osteosarcoma. (DOCX 1962 kb) [file 13045_2018_560_MOESM2_ESM.docx]

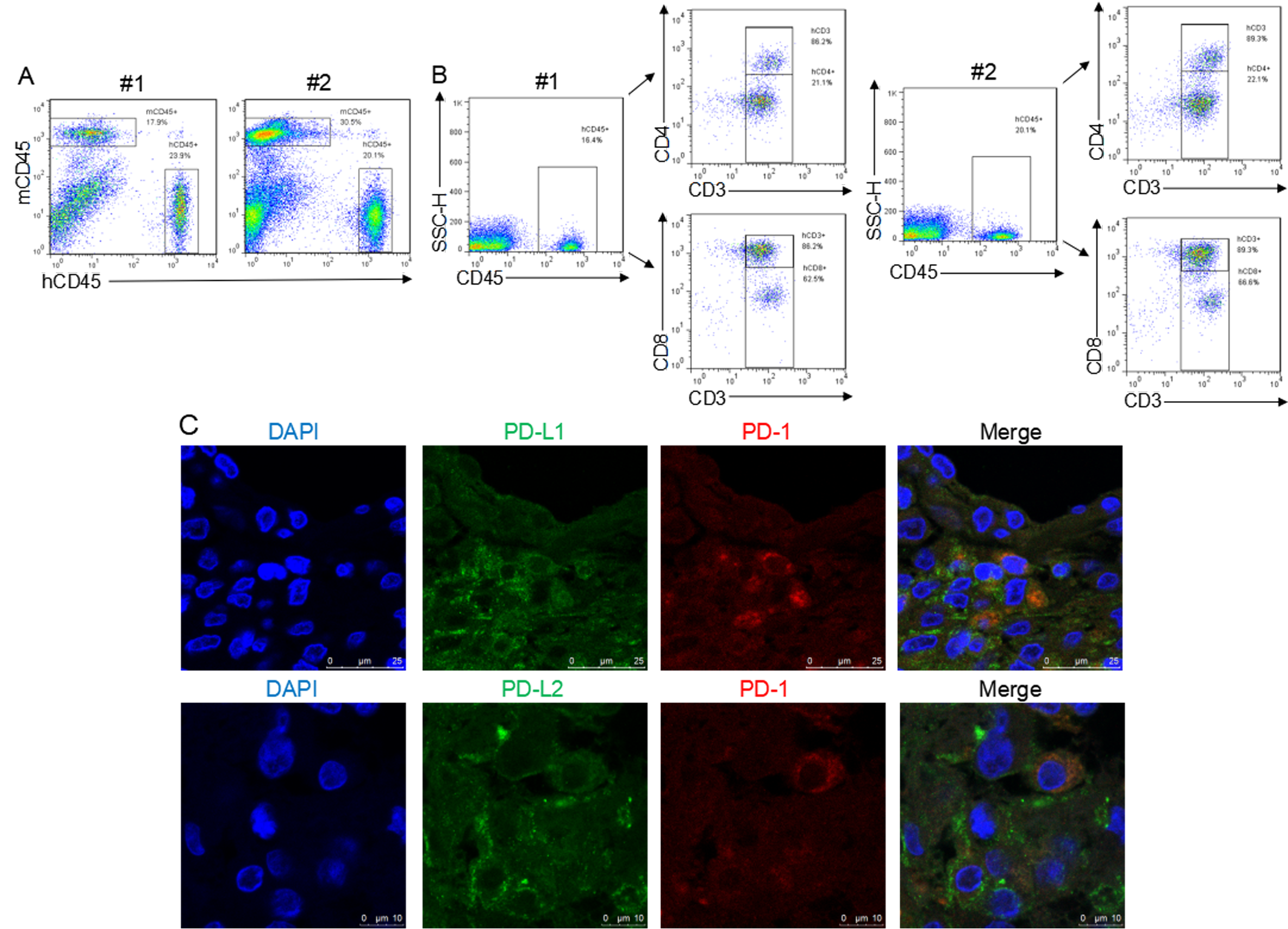


Figure S1. (A) Representative images of the assessment of the human CD45 positivity cell rate in humanized mice by flow cytometry; the positivity rates were greater than 25%. (B) The proportion of the human CD4- and CD8-positive cells to the human CD45-positive cells was also determined by flow cytometry. As the representative images show, most of the human CD45-positive cells are lymphocytes. (C) Double immunofluorescence staining indicated the colocalization of PD-L1/PD-1 and PD-L2/PD-1.
