# Supplementary material for: PD-1 axis expression in musculoskeletal tumors and antitumor effect of nivolumab in osteosarcoma model of humanized mouse
Source: J Hematol Oncol. 2018 Feb 6;11:16. doi: 10.1186/s13045-018-0560-1 (PMC5801803; doi:10.1186/s13045-018-0560-1)
Supplement: Supplementary file 3 — PD-L1, PD-L2 and PD-1 expressions in osteosarcoma. (DOCX 1426 kb) [file 13045_2018_560_MOESM3_ESM.docx]

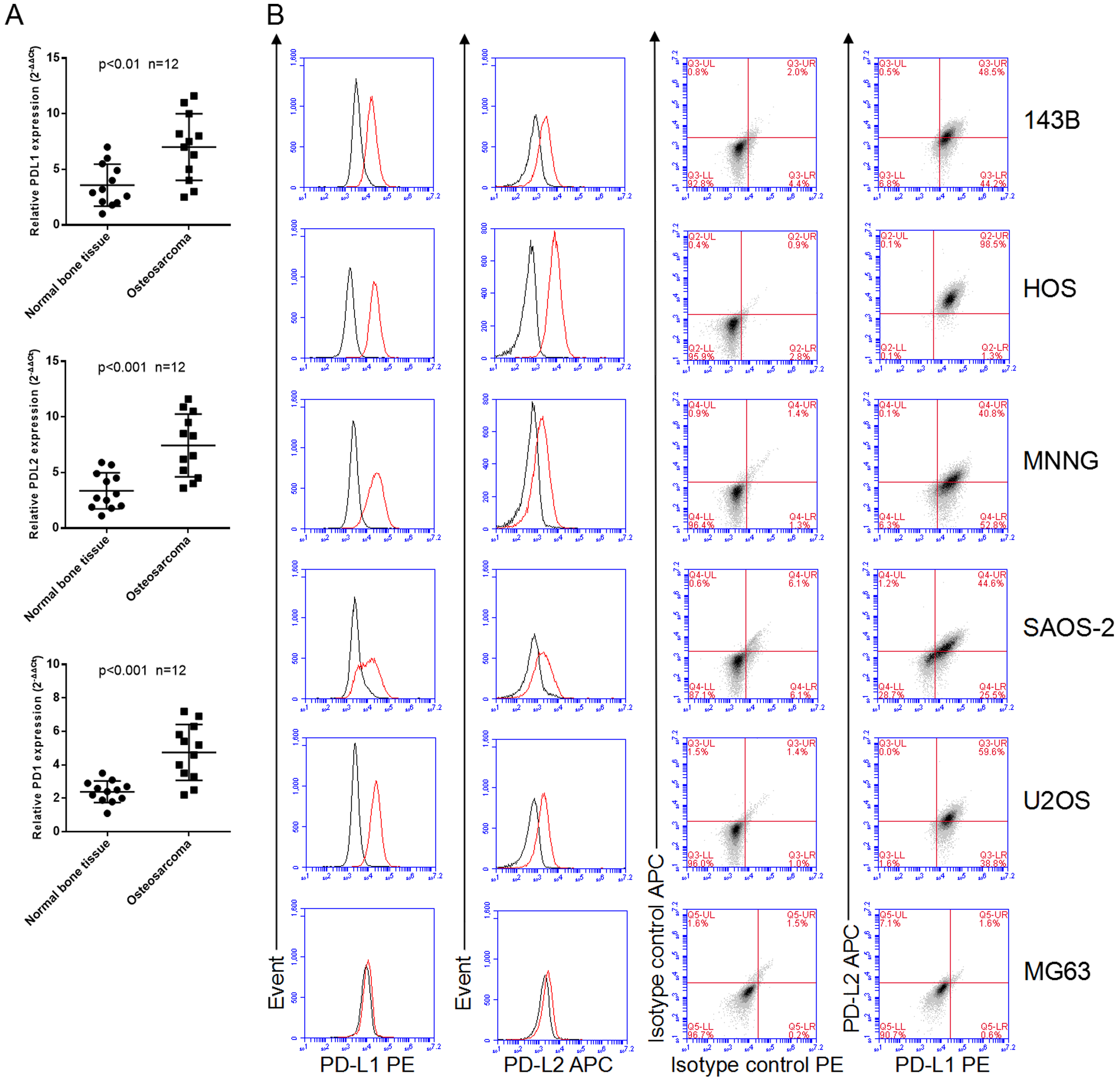


Figure S2. PD-L1, PD-L2 and PD-1 expressions in osteosarcoma. (A) PD-L1, PD-L2 and PD-1 mRNA expressions were upregulated in osteosarcoma tissues. (B) The osteosarcoma cell lines (red) exhibited differing degrees of membranous expression of PD-L1 and PD-L2 compared with those in the isotype control (black) by flow cytometry. Data are presented as the mean±S.D. **P＜0.01, ***P＜0.001 by Student’s t-test.
